# Reconstructing high-dimensional two-photon entangled states via compressive sensing

**DOI:** 10.1038/srep06542

**Published:** 2014-10-13

**Authors:** Francesco Tonolini, Susan Chan, Megan Agnew, Alan Lindsay, Jonathan Leach

**Affiliations:** 1 SUPA, School of Engineering and Physical Sciences, Heriot-Watt University, Edinburgh EH14 4AS, UK; 2 Applied and Computational Mathematics and Statistics, University of Notre Dame, USA

## Abstract

Accurately establishing the state of large-scale quantum systems is an important tool in quantum information science; however, the large number of unknown parameters hinders the rapid characterisation of such states, and reconstruction procedures can become prohibitively time-consuming. Compressive sensing, a procedure for solving inverse problems by incorporating prior knowledge about the form of the solution, provides an attractive alternative to the problem of high-dimensional quantum state characterisation. Using a modified version of compressive sensing that incorporates the principles of singular value thresholding, we reconstruct the density matrix of a high-dimensional two-photon entangled system. The dimension of each photon is equal to *d* = 17, corresponding to a system of 83521 unknown real parameters. Accurate reconstruction is achieved with approximately 2500 measurements, only 3% of the total number of unknown parameters in the state. The algorithm we develop is fast, computationally inexpensive, and applicable to a wide range of quantum states, thus demonstrating compressive sensing as an effective technique for measuring the state of large-scale quantum systems.

Many areas of quantum mechanics require the efficient and accurate measurement of entangled states. Perhaps the most traditional and widely adopted way of doing so is through full tomographic reconstruction^1^, a technique that performs a series of independent measurements on the system in order to uniquely identify its nature. However, the complexity of such a method dramatically increases with increasing dimension of the system, and fully measuring the state of two entangled objects, each of *d* dimensions, requires at least *d*^4^ measurements^2^. As a result, full tomographic reconstruction is effective only at low dimensions and is otherwise prohibitively time consuming and computationally expensive.

Large-dimensional states are necessary for quantum computation and for certain quantum information protocols. Monz *et al.* reported the generation of a 14-qubit entangled state using trapped ions^3^, and Yao *et al.* reported the generation of an 8-photon entangled state^4^, although neither reported the density matrix for their respective states. Zhang *et al.* performed quantum tomography of a hybrid optical detector with over a million free parameters^5^. However, to date, the largest density matrix reported for an entangled state is that of Häffner *et al.*, who recorded the density matrix of 8 trapped ions^6^.

Compressive sensing, which originates from the field of signal processing, provides a very efficient mechanism to establish properties of an unknown system with limited observations (see, e.g., Candès^7^ and references therein). Compressive sensing uses prior assumptions in order to reduce the number of possible solutions, which can drastically reduce both measurement and processing time. Consequently, it is possible to establish descriptions of very large systems that could previously not be explored. This principle is used extensively in the fields of image reconstruction^8^ and medical tomography^9^, and it has recently been adopted in various areas of quantum science^10^,^11^,^12^,^13^,^14^,^15^,^16^.

In this paper we propose and outline a compressive sensing technique that is able to successfully reconstruct the density matrix of near-pure entangled states of high dimensions. We implement this method to reconstruct a pure state of two 17-dimensional photons entangled in their orbital angular momentum. The recovery of the state is achieved by employing only 3% of the measurements that full tomographic reconstruction would require. The full procedure, including measuring and post-processing, takes approximately three hours on a standard desktop computer. Our data processing algorithm is similar to the singular value thresholding algorithm detailed in^17^; however, its design is specifically adapted for near-pure entangled state reconstruction. The procedure is fast, computationally inexpensive, and robust to noise.

## Results

### Theoretical description of compressive sensing and quantum tomography

Compressive sensing is a data-processing technique widely used in different signal reconstruction applications. Its aim is to find the solution to underdetermined linear systems, under the assumption that such a solution is sparse in some basis. Such problems can be posed in the following way: 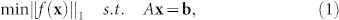
where 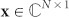 represents a vector describing the measured object; 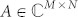 is the matrix of measurements, with 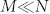; 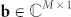 is the vector of measurement results; || **·** ||_1_ denotes the *ℓ*1 norm of the vector; and *f* is a transformation to a space in which *f*(**x**) has a sparse representation.

In the specific case of quantum state tomography, the aim is to reconstruct an unknown near-pure density matrix, using an under-sampled set of measurements, under the assumption that such a matrix is low rank. The problem to be solved is then^10^,^18^

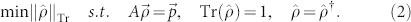
Here, 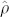 is the density matrix to be reconstructed, while 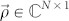 is the density matrix in vector form; 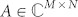 is the matrix of measurements; 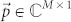 is the vector of resulting probabilities; and || **·** ||_Tr_ stands for the trace norm of the matrix. The rows of the measurement matrix *A* are individual measurement vectors *A_i_*, and the elements of the vector 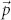 are the corresponding probabilities *p_i_*.

### The algorithm

We develop an operation-projection method similar to the singular value thresholding algorithm shown in^17^ and implemented in^10^; however, we significantly modify its design in order to make use of the known features of near-pure entangled states. While singular value thresholding relies on both decomposing and recomposing the matrix using singular value decomposition, our method instead recomposes the matrix using the assumption that it is Hermitian. The algorithm requires an initial guess matrix to begin the procedure. The protocol then has two main stages: (i) the operations on the current matrix 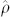 to impose the desired characteristics and (ii) the projection of the resulting answer in vector form 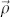 onto the solution space. Applying these steps repeatedly constitutes an iterative procedure to approach the target solution. We interchange between the matrix form and vector form when implementing the operation and projection stages respectively.

In the operations stage, two steps are performed: First, the rank of the matrix is reduced by thresholding the eigenvalues below a certain level. This is achieved by decomposing the matrix into its eigenvalues and eigenvectors, setting the eigenvalues below the chosen threshold to zero, and then recomposing the matrix using 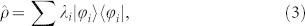
where *λ_i_* is the *i^th^* eigenvalue and *φ_i_* the corresponding eigenvector. Second, to make use of the known sparsity characteristics associated with entangled states, we apply a thresholding operation on the individual matrix elements. We achieve this by setting the elements that have modulus smaller than a chosen value to zero. To apply the method to a state that is not known to be entangled, this step can be excluded. Finally, we normalise the result to have trace equal to unity to obtain a density matrix.

After the operation, the resultant matrix 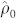 has the desired characteristics of the solution; however, it no longer belongs to the linear space defined by the measurements *A* and probabilities 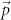. To return the matrix 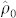 to the space defined by 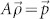, we then implement the projection stage of the procedure. In order to describe the projection stage, we first introduce a geometrical formalism of the problem.

**Figure 1:**
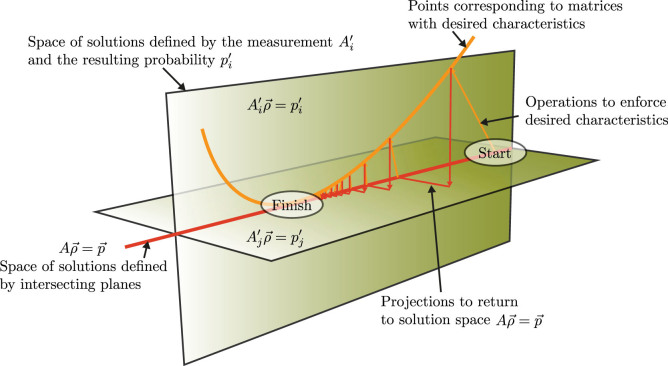
A schematic representation of the compressive sensing problem. The two green planes 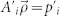 and 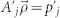 each correspond to individual measurements and represent two different solution spaces. The intersection of the two planes corresponds to the set of all solutions belonging to the combined space 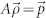, indicated by the red line. The curved line represents a set of potential solutions in the space that retain the desired characteristics of our answer. Our algorithm works by iterating between the set of solutions with the desired characteristics (orange line) and the set of solutions belonging to 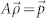 (red line). After a number of iterations, the algorithm converges to the solution of 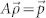 that possesses the desired characteristics.

Each measurement vector *A_i_* and corresponding probability *p_i_* represents a hyperplane in a space of *N* dimensions, where *N* is the number of elements in 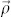. This can be understood by visualising each measurement as the normal to a plane; the probability resulting from the measurement provides the intercept of the plane with the normal, which completely defines a plane in which the solution can reside. The intersection of these hyperplanes represents the set of all solutions to the system 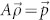. A simplified version of this concept is shown in Fig. 1, where two intersecting hyperplanes are represented as two-dimensional planes, and their intersection as a line.

After the operations stage, the matrix 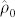 is reshaped into vector form 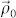 so that it can be projected onto the intersection of the hyperplanes defined by the linear system. The projection procedure is simple and computationally inexpensive if the hyperplanes are all perpendicular to each other.

However, although the matrix of random measurements *A* is nearly orthonormal, there is small non-zero overlap between any two measurements *A_i_* and *A_j_* (*i* ≠ *j*). This is due to the physical limitations of the measurement procedure. For this reason, we transform the system 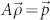 into a new system 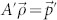, where *A′* is an orthogonal matrix. This is achieved by multiplying both *A* and *p* by a matrix *B* such that *BA* = *A′*. It is important to note that the system 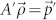 is a mathematical construct and no longer directly relates to the measurements and their corresponding probabilities; however, the set of solutions it defines is exactly the same as that of the original system.

In order to obtain a solution 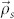 from the initial point 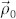, we progressively project 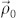 on each hyperplane in turn. This procedure is initiated by projecting the initial point 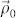 onto the first hyperplane, given by 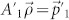, to find a new point 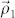. This new point is then projected onto the second hyperplane, and we continue in this fashion until the desired solution 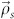 is found. This occurs after *M* projections, where *M* is the number of measurements. Details of this projection procedure can be found in the Supplementary Materials.

Applying this operation-projection procedure repeatedly constitutes an iterative method that provides a solution exhibiting the desired characteristics and belongs to the linear system 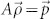. The schematic outline of the algorithm is shown in Fig. 1, where the orange and red arrows represent the operation and projection steps, respectively. The method is considered complete when the distance between consecutive iterates is below a predetermined tolerance.

### Noise correction

In our system, noise manifests itself as errors in the measured probabilities. Such noise is unavoidable, and consequently, the density matrix that we recover 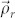 will not correspond to the desired solution to the problem 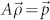; instead, it will be a solution to the system 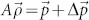, where 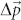 is a vector of errors on the true probabilities. This error in probabilities results in a solution 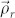 that is in fact some distance 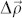 from the desired solution 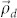 in the space in which the algorithm operates. There are many methods for finding the solution in the presence of error^17^,^19^. In our case, we determine 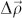 and subtract it from 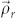. This corresponds to the operation 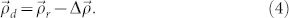
We use *a priori* knowledge of the desired state's characteristics to find 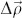 and systematically correct for noise in the system. Further details of our method can be found in the Supplementary Information.

### Experimental implementation

**Figure 2:**
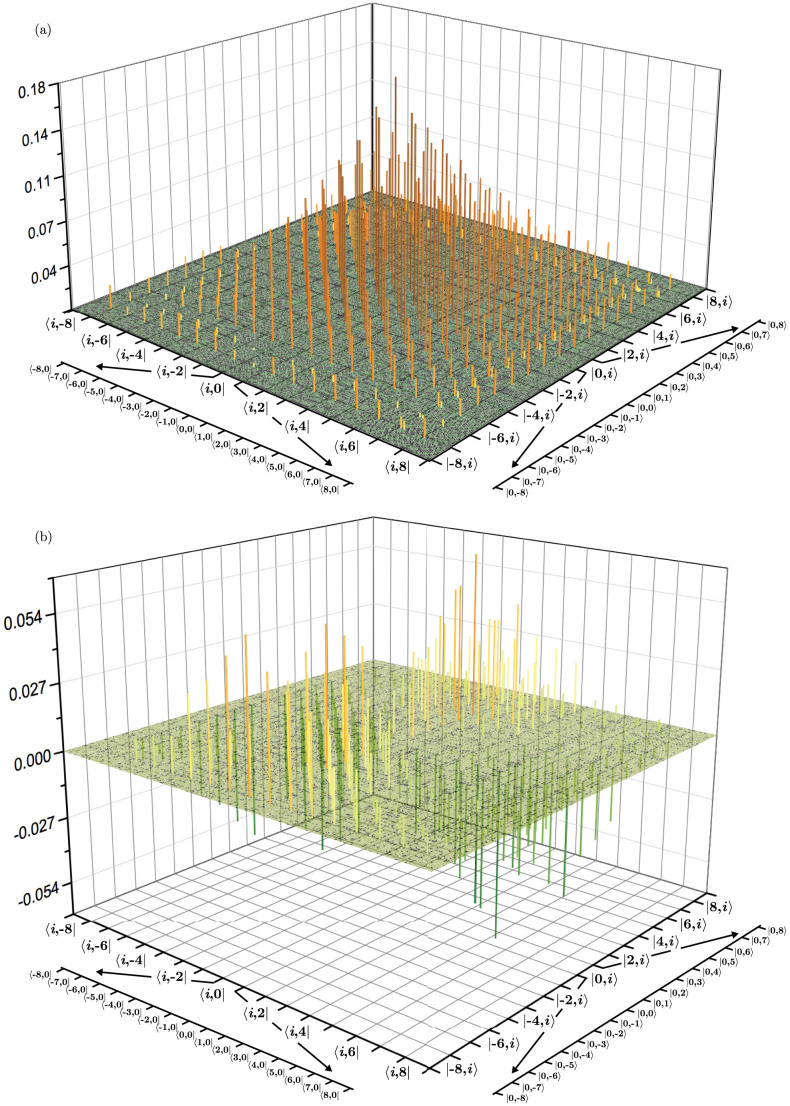
(a) The real part of the recovered density matrix. The dimension of each photon is equal to *d* = 17, so that the number of unknown parameters of the combined space is equal to 83521. The index *i* runs from *i* = −8 to 8. (b) The imaginary part of the recovered density matrix.

We have performed an experimental recovery of the density matrix of a 17-dimensional two-photon state in the orbital angular momentum (OAM) degree of freedom, produced by parametric downconversion (see Methods for details). The dimension of each photon is equal to *d* = 17, so the number of unknown parameters in the entire state is 83521. The reconstruction is performed after 2506 projective measurements, which corresponds to only 3% of the total number of unknown parameters in the state. The reconstruction of the state is shown in Fig. 2.

The state that we measure exhibits strong anti-correlations in the OAM degree of freedom; that is to say that the OAM state |*ℓ*〉*_S_* in the signal photon is correlated with the state |−*ℓ*〉*_I_* in the idler photon. Additionally, the existence of the non-zero off-diagonal elements in the density matrix indicates a high degree of purity in the obtained state. These two features combine to suggest a high degree of entanglement of the OAM modes.

To characterise the entanglement, we use the fidelity of the reconstructed state *ρ* with the ideal, maximally entangled pure state 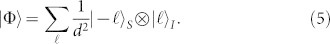
The fidelity is then given by 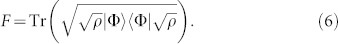
For the density matrix shown in Fig. 2, this fidelity was found to be 83.1%.

**Figure 3:**
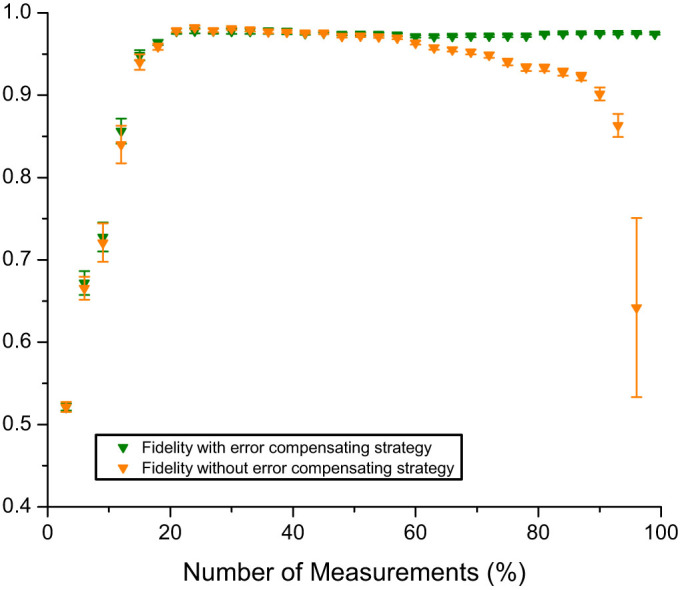
Fidelity with the maximally entangled state vs. the percentage of measurements used for reconstruction for dimension *d* = 7 (100% corresponds to *d*^4^ random projective measurements). The orange points correspond to the fidelities without error compensation; the green points correspond to the fidelities with our error compensation. The maximum value for the green points is 0.97 ± 0.01.

In order to characterise the effectiveness of the reconstruction method, we reconstructed the density matrix of a 7-dimensional two-photon state with varying number of measurements. The resultant fidelities are shown in Fig. 3. We show the results both with and without our error correction procedure. For both cases, the fidelity increases as the number of measurements increases, indicating that more information produces a more accurate reconstruction.

However, for the case without error correction, the fidelity gradually decreases beyond 20% of the measurements. Because the measurements performed are nearly orthogonal to each other and are of insufficient number to yield a fully determined system, the errors contained within each measurement result do not average out to reduce the uncertainty, but instead sum to increase the uncertainty. Equivalently, every measurement taken into account restricts by one dimension the space of possible solutions to the underdetermined system: fidelity increases with increasing measurements at a low number of samples because the space is large enough to be very close to the desired solution, but the space gets smaller with increasing measurements, progressively excluding other low-rank sparse objects. At the high fidelity peak, the space is small enough so that the lowest rank and sparsest solution it contains is approximately the desired one and the algorithm will converge towards it. As the number of samples increases, the accumulation of errors results in a solution space that is far from the desired one; however, with additional samples, the dimension of the space is reduced. As a result, its distance from the sampled object increases and the algorithm yields an answer that diverges from the desired one.

## Discussion

We have developed and tested an efficient method for determining the state of a quantum system based on a few simple assumptions. In this case, we use the prior knowledge of the sparsity of the density matrix associated with the system to achieve high-fidelity recovery from a small number of independent measurements of that system. Thus the state that we report corresponds to that which satisfies the set of measurements and the initial assumption of purity. One way to look at this is to say that we have answered the following question: “What is the purest state that is compatible with the set of measurements?” However, a feature of our method is that, using the same measurements and different prior knowledge, it can be readily refashioned to recover many states with a variety of desired characteristics.

In the case of a two-photon entangled state, where each photon exists in a 17-dimensional space with 83521 corresponding unknowns, we are able to recover the system with 3% of the measurements required for informational completeness of an unknown general quantum state. Our result corresponds to one of the largest discrete quantum states yet to be reported. We anticipate that the techniques implemented in this work will have impact in a wide range of areas in quantum science, including implementation and verification of quantum information protocols using high-dimensional states.

## Methods

**Figure 4:**
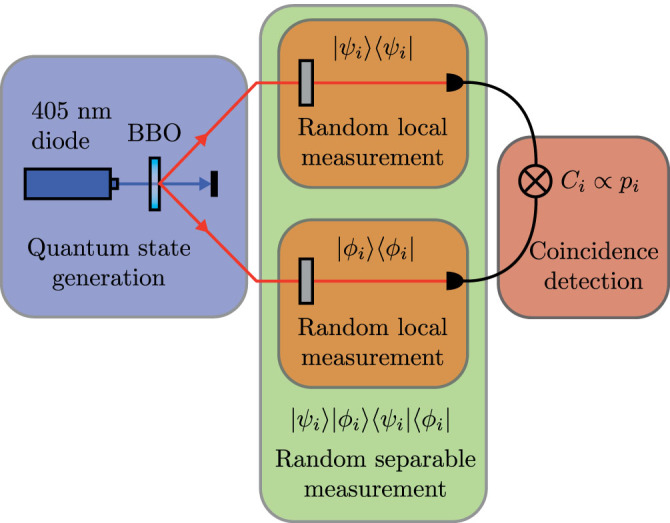
Experiment configuration for compressive sensing of high-dimensional quantum states entangled in the orbital angular momentum degree of freedom.

We use a 100-mW diode laser with wavelength 405 nm, along with a 3-mm-thick BBO crystal, to generate entangled photons through the process of parametric downconversion; see Fig. 4. The two-photon state that is generated in this process is given by 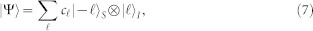
where |*c_ℓ_*|^2^ indicates the probability of finding the signal photon with 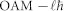 and the idler photon with OAM 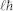. In our experiment, we limit the range of OAM states to values between *ℓ* = −8 and *ℓ* = 8.

The signal and idler photons are each incident on a separate half of a spatial light modulator (SLM), displaying computer-generated holograms, and then collected by a single-mode fibre connected to a single-photon detector. This results in a projective measurement on the two-photon mode. The result of the projection is measured by the coincidence detection with a coincidence window of 25 ns. Each measurement is performed for 3 s with a maximal coincidence rate of approximately 450 counts/sec.

One of the keys to successful compressive sensing is to ensure that the measurement states are unstructured with respect to the basis in which the sampled state is sparse. For this experiment, that corresponds to measurement settings that are random superpositions of OAM modes. Therefore the measurement states |*ψ_i_*〉*_S_* and |*ϕ_i_*〉*_I_* are generated from superpositions of OAM states where the coefficients *a_ℓ_* are generated at random 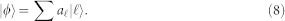
We generate the matrix *A*, of Eq. (2), by performing a number of random separable projective measurements. Each row of *A* corresponds to the vector form of the individual projectors 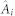. The projection operator 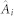 is given by 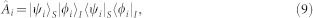
where the states |*ψ_i_*〉*_S_* and |*ϕ_i_*〉*_I_* are the modes for the signal and idler arms respectively.

The coincidence rate *c_i_* for each measurement 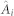 can be normalised to obtain the equivalent probability *p_i_*. Each probability *p_i_* constitutes the result of the corresponding measurement 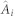. The probability of recording a coincidence count is given by 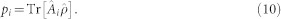
Consequently, the linear system that is defined by the set of measurement operators 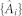 and the corresponding probabilities {*p_i_*} is 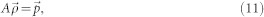
where 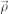 is the vector form of the density matrix 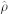. After performing an appropriate number of measurements, the task is then to solve the inverse problem under the constraints given by Eq. (2).

## Author Contributions

J.L. and A.L. conceived the project, F.T., S.C. and M.A. performed the experiment and data analysis, F.T., M.A. and J.L. wrote the main manuscript text and F.T. and J.L. prepared figures 1–4. All authors contributed to the final version of the manuscript.

## Supplementary Material

Supplementary Information Supplementary Information
