# Supplementary material for: Reconstructing high-dimensional two-photon entangled states via compressive sensing
Source: Sci Rep. 2014 Oct 13;4:6542. doi: 10.1038/srep06542 (PMC4194436; doi:10.1038/srep06542)
Supplement: Supplementary Information [file srep06542-s1.pdf]

Reconstructing high-dimensional two-photon entangled states via compressive sensing

Francesco Tonolini[1], Susan Chan[1], Megan Agnew[1], Alan Lindsay[1,2], Jonathan Leach[1,*]
[1] *SUPA, School of Engineering and Physical Sciences, Heriot-Watt University, Edinburgh EH14 4AS, UK*
[2] *Applied and Computational Mathematics and Statistics, University of Notre Dame, USA and*
[*] *j.leach@hw.ac.uk*

SUPPLEMENTARY MATERIALS

Algorithm Schematics

Input: Matrix of measurements $A \in \mathbb{C}^{M\times N}$, vector of normalised probabilities $\vec{p}$, initial guess matrix $\hat{\rho}_{in}$, SVD threshold parameter τ such that $0 < \tau < 1$, sparsity parameter τ_ℓ such that $0 < \tau_\ell < 1$ and stopping condition step size δ_s. In our 17-dimensional state reconstruction we choose $\tau = 0.4$, $\tau_\ell = 0.04$ and $\delta_s = |\vec{\rho}_s| \cdot 10^{-3}$.
Output: Recovered matrix $\hat{\rho}_d$

1. Set $A' = orth(A)$ and $\vec{p}\,' = C\vec{p}$ where $CA = A'$
2. Set $\hat{\rho}_{s,0} = \hat{\rho}_{in}$
3. **For** $k = 1 : k_{max}$
4. Set $\hat{\rho}_{0,k} = \Gamma_{\tau,\tau_\ell}(\hat{\rho}_{s,k-1})$
5. Set $\vec{\rho}_{0,k} = vec(\hat{\rho}_{0,k})$
6. **For** i=1:M
7. Set $A_i = i_{th}$ row of A
8. Set $\vec{\rho}_i = P(\vec{\rho}_{i-1}, A_i)$
9. **End**
10. Set $\hat{\rho}_{s,k} = mat(\vec{\rho}_M)$
11. Set $\delta = |\hat{\rho}_{s,k} - \hat{\rho}_{s,k-1}|$
12. **If** $\delta \le \delta_s$ **Break**
13. **End**

Here $orth(\cdot)$ is the orthogonalizing operator, $\Gamma_{\tau,\tau_\ell}(\cdot)$ is the operator that enforces the desired characteristics described in the results section, $vec(\cdot)$ is the operator that rearranges the elements of a matrix into a vector, $P(v, V)$ denotes the projection of a vector v onto a hyperplane having normal V, and $mat(\cdot)$ is the operator that rearranges the elements of a vector into a square matrix.

Projection onto a hyperplane

To project a point $\vec{\rho}_{i-1}$ onto a hyperplane $A'_i\vec{\rho} = p'_i$, it is necessary to find the vector $\vec{v}_i$ that has direction $\vec{n}_i$ normal to the hyperplane $A'_i\vec{\rho} = p'_i$ and size k_i, where k_i is

$$k_i = p'_i - \langle \vec{n}_i | \vec{\rho}_{i-1} \rangle. \tag{1}$$

The desired projection $\vec{\rho}_i$ is then

$$\vec{\rho}_i = \vec{\rho}_{i-1} + \vec{v}_i. \tag{2}$$

Finding and correcting $\Delta\vec{\rho}$

The error vector $\Delta\vec{\rho}$ depends on the experimental error Δp_i associated with each of the probabilities and the measurements made to perform the reconstruction. Instead of extending the search to a non-linear space, we make use of the low rank and sparsity information to estimate the error direction, that is, to find a close approximation for the vector $\Delta\vec{\rho} = \vec{\rho}_r - \vec{\rho}_d$, where $\vec{\rho}_r$ is the projection of $\vec{\rho}_d$ onto the linear space intersection of all the hyperplanes. In order to do this we divide the measurements and the corresponding probabilities in subsets A_{s_i} and $\vec{p}_{s_i}$ of sufficient size for our compressive sensing technique to yield convergence to a solution (the size of the subsets depends on the purity of the state and the estimate of the error Δp_i on each of the probabilities). We then perform the operation-projection algorithm on each subset separately to find the vectors $\vec{\rho}_{s_i}$, low rank and sparse solutions to the corresponding subsets systems $A_{s_i}\vec{\rho} = \vec{p}_{s_i}$. We hence define a new set of vectors $\Delta\vec{\rho}_i = \vec{\rho}_{s_i} - \vec{\rho}_{d_i}$ where $\vec{\rho}_{d_i}$ is the solution resulting from applying one last set of thresholding operations to $\vec{\rho}_{s_i}$. The sum of the vectors $\Delta\vec{\rho}_i$ is taken as a close approximation for the error vector $\Delta\vec{\rho}$. We finally compute a correction vector for the probabilities Δp that can be subtracted from the measured probabilities p. The correction vector is defined as

$$\Delta p = A\Delta\vec{\rho}. \tag{3}$$

Once the probabilities have been corrected as described above, the compressive sensing algorithm can be performed, making use of the set of performed measurements A and the corrected probabilities $p - \Delta p$.
